# Supplementary material for: Mechanisms governing the pioneering and redistribution capabilities of the non-classical pioneer PU.1
Source: Nat Commun. 2020 Jan 21;11:402. doi: 10.1038/s41467-019-13960-2 (PMC6972792; doi:10.1038/s41467-019-13960-2)
Supplement: Supplementary file 7 — Source data [file 41467_2019_13960_MOESM7_ESM.zip › Source_Data/FigureS2/FigureS2B_MotifScanOutput/KmeansCluster1/homerResults/motif22.similar.html]

motif22

## Information for motif22

C
T
A
G
C
G
T
A
T
C
A
G
A
G
C
T
G
T
A
C
A
G
C
T
A
C
G
T
A
G
C
T
C
G
T
A
C
T
A
G
C
T
A
G
C
A
T
G
G
A
C
T
  
Reverse Opposite:  

C
T
G
A
G
T
A
C
A
G
T
C
G
A
T
C
A
C
G
T
C
T
G
A
C
G
T
A
C
T
G
A
A
C
T
G
T
C
G
A
A
G
T
C
C
G
A
T
A
G
T
C
  

|  |  |
| --- | --- |
| p-value: | 1e-38 |
| log p-value: | -8.847e+01 |
| Information Content per bp: | 1.771 |
| Number of Target Sequences with motif | 59.0 |
| Percentage of Target Sequences with motif | 1.16% |
| Number of Background Sequences with motif | 20.2 |
| Percentage of Background Sequences with motif | 0.05% |
| Average Position of motif in Targets | 91.5 +/- 54.9bp |
| Average Position of motif in Background | 124.2 +/- 57.5bp |
| Strand Bias (log2 ratio + to - strand density) | 0.2 |
| Multiplicity (# of sites on avg that occur together) | 1.00 |
| Motif File: | file (matrix) reverse opposite |

### Similar de novo motifs found

|  |  |  |  |  |  |  |  |
| --- | --- | --- | --- | --- | --- | --- | --- |
| Rank | Match Score | Redundant Motif | P-value | log P-value | % of Targets | % of Background | Motif file |
| 1 | 0.942 | C T G A C T A G A C G T G T A C G A C T A C G T G A C T G T C A C A T G C A T G A T C G A G C T | 1e-32 | -75.323839 | 1.02% | 0.05% | motif file (matrix) |
| 2 | 0.930 | G T A C G A T C C G A T C T G A G T C A T G C A C A T G C G T A A G T C A C G T A G T C | 1e-30 | -69.465088 | 1.53% | 0.22% | motif file (matrix) |
| 3 | 0.888 | T G A C G T A C C G A T C G T A C G T A G T C A A C T G C G T A A G T C A C G T | 1e-19 | -45.167854 | 1.34% | 0.28% | motif file (matrix) |
| 4 | 0.845 | C G T A A C T G A C G T A G T C A C G T A C G T A C G T C G T A A C T G | 1e-13 | -31.733343 | 0.77% | 0.13% | motif file (matrix) |
